# Selective recovery of copper from copper tailings and wastewater using chelating resins with bis-picolylamine functional groups

**DOI:** 10.1016/j.heliyon.2024.e27766

**Published:** 2024-03-12

**Authors:** Kosisochi Ibebunjo, Youssef El Ouardi, John Kwame Bediako, Anna Iurchenkova, Eveliina Repo

**Affiliations:** a School of Engineering Science, Department of Separation Science, LUT University, FI-53850, Lappeenranta, Finland; b Uppsala University, Disciplinary Domain of Science and Technology, Technology, Department of Materials Science and Engineering, Nanotechnology and Functional Materials, Sweden

**Keywords:** Chelating resin, Bis-picolylamine, Copper, Selectivity, Adsorption, Recovery

## Abstract

Industrial and mining wastewater, along with copper tailings, are typically highly acidic and contain copper and other heavy metals, which may contaminate and damage the environment. Copper (Cu) is, however, a valuable metal, making its removal and recovery from such wastewater and tailings environmentally and economically advantageous. Chelating ion exchange resins featuring bis-picolylamine functional groups are especially suitable for application requiring selective recovery of Cu(II) from highly acidic media. In this study, and for the first time, the kinetics, binding capacity and selectivity of Lewatit MDS TP 220 chelating resin towards Cu(II) are reported. The resin was characterized by Zeta potential, scanning electron microscope (SEM), Fourier-transform infrared spectroscopy (FTIR), and X-ray photoelectron spectroscopy (XPS). Factors including pH, initial concentration, contact time, temperature, and selectivity were investigated to assess the adsorption performance of the chelating resin. The adsorption kinetics tests revealed fast adsorption within the first 5–30 min and fitted the pseudo-second-order model, signifying chemisorption process. The adsorption isotherm followed the Langmuir model, implying monolayer adsorption process. The maximum adsorption capacity (*q*_*m*_) for Cu(II) determined by the Langmuir model was 103.9 mg/g. The adsorption thermodynamics showed an endothermic and spontaneous adsorption. FTIR and XPS studies suggested coordination or chelation as the possible adsorption mechanism. Lewatit MDS TP 220 exhibited excellent Cu(II) adsorption, desorption with 2 M ammonium hydroxide (NH_4_OH), and selectivity in multi-metal ions solution. Additionally, the resin demonstrated excellent reusability after five regeneration steps. This chelating resin is a potential adsorbent for effective and recurrent recovery of Cu(II) from copper tailings and wastewater, thereby contributing to environmental remediation.

## 1 Introduction

Effectively separating heavy metals from aqueous solutions addresses several practical concerns such as remediation of heavy metal contaminated wastewater and the recovery of valued metals [1]. Water bodies become contaminated with heavy metals mainly due to the release of industrial and mining effluents characterized by high concentrations of these metals [2]. Owing to their inherent non-biodegradability, these metals often accumulate and persist within aquatic environments for extended periods of time, thereby posing significant hazards to humans, animals, and plants. Copper (Cu) is a heavy metal that is commonly found in elevated levels in wastewater because it is extensively used in a variety of industries, either as a raw material or as an additive [2,3]. Cu is required for plant, human, and animal growth and development. Nevertheless, excessive consumption can have adverse effects including diabetes, Wilson's disease, cancer, and even death in humans. Similarly, plants may experience hindered growth, less branches, and roots decay [4]. Hence, remediation of wastewater contaminated with copper before its release is crucial as even trace amounts of copper is environmentally hazardous [2,3].

On the other hand, Cu has significant uses and is a key component of raw materials and goods for human use and wellbeing. It is used in numerous branches of industry such as mining, refining, construction, architecture, transportation, pipe systems, energy, electrical systems, healthcare, general consumer items, among others [2,[4], [5], [6], [7]]. As a result, production of Cu has escalated from about 600,000 tonnes to 850 million metric tonnes between 2010 and 2021, representing a yearly average growth rate of more than 4% [6]. Copper mining generates a significant byproduct known as tailings, or Cu tailings [[8], [9], [10]]. Copper tailings (CTs) constitute 36.7 % of the global annual tailings production, which amounts to 3.44 million metric tonnes [11,12]. They contain residues of Cu and additional metals that remained unrecovered during the extraction process. These residues are usually accumulated in tailings reservoirs without subsequent processing or application, resulting in their leaching into adjacent surroundings, thereby causing contamination and considerable ecological damage [9,10,13]. Owing to the extensive applications of Cu coupled with the increasing production, various tailings and wastewaters containing this metal are produced [4].

Since Cu is an essential and beneficial metal, removing, and recovering it from wastewater and CTs even in trace amount, will be economically beneficial for metal recycling and also address significant environmentally concerns. However, Cu coexists with various heavy metal ions in wastewater and tailings, and selective removal and recovery is important for potential reuse [3]. Moreover, wastewater originating from most industries are strongly acidic because of the large concentration of acids used in these industries. Their treatment is seldom studied compared to weakly acidic or neutral wastewater because they require neutralization before treatment which results in significant alkali consumption, need for further sediment treatment, and waste of useable inorganic acid [1,14]. As a result, efficient and cost-effective ways to recover Cu selectively from very acidic wastewater and tailings are required.

Numerous investigations have reported the selective Cu(II) removal and recovery from aqueous solutions [[2], [3], [4],^6^,^7^,^15^,^16^] and the unique and high affinity of copper towards chelating polymers possessing solely nitrogen donors in strongly acidic conditions has also been reported. Cu(II) is characterized as a moderately soft Lewis acid while the nitrogen donors such as secondary or tertiary amines exhibit characteristics of soft Lewis bases [14]. Therefore, chelating ion exchange resins possessing bis-picolylamine functional groups are well-suited for applications where selective recovery of Cu(II) from highly acidic media (pH < 2) is required [1,6,14]. These resins have good acid resistance because of their picolylamine-based structure [1]. Bis-picolylamine functional groups feature three nitrogen donors, two of which are located in the pyridine rings. At very low pH (<2), these nitrogen donors remain deprotonated due to their low pKa values, which is facilitated by the electron-withdrawing influence of the aromatic group [1,14,17]. Consequently, under the most acid condition, all the nitrogen donors are protonated. Within moderately acidic pH levels, one of the nitrogen donors within the pyridine becomes deprotonated, while at pH levels greater than 3.5, all nitrogen donors deprotonate, and become available for the binding of metal ions.

Prior research has showcased the removal and recovery of Cu(II) using chelating resins containing this functional group [14,17]. Neto et al. employed Dowex M 4195 (M 4195) resin to recover 99% of Cu(II) selectively from electronic waste solution that contained various heavy metals. Utilizing the pseudo-second-order model proved optimal for depicting the adsorption kinetics, suggesting that chemisorption was the prevailing mechanism. Additionally, the Langmuir model proved to be the most suitable for depicting the adsorption isotherm, indicating monolayer adsorption of Cu(II) onto the sorbent. Moreover, Kołodyńska et al. compared M4195 to Lewatit Monoplus TP 220 (TP220) and found out that TP220 was more effective, with 94.20 mg/g as its maximum adsorption capacity (q_m_) as opposed to M4195, with a *q*_*m*_ of 56.66 mg/g. Here also, the kinetics and isotherm followed the same mechanisms as with M4195 [14].

Lewatit MDS TP 220 is a chelating resin similar to Lewatit Monoplus TP 220 except that it has a small bead size and monodisperse bead size distribution, which results in quicker adsorption kinetics and 25% greater Cu(II) binding capacity. However, to our knowledge, no reports have been made regarding the kinetics and Cu(II) binding capacity of this resin. This work therefore investigates the Cu(II) binding capacity and selectivity of Lewatit MDS TP 220 in batch adsorption mode. Several factors including the pH, initial concentration of metal ion, contact time, temperature, and selectivity in the presence of other metal ions were executed to understand their effect on Cu(II) removal. The recovery of Cu(II) and the reusability of Lewatit MDS TP 220 was investigated, including the adsorption kinetics and equilibrium isotherms.

## 2 Materials and methods

### 2.1 Materials

Lewatit MDS TP220 (LTP) was supplied by Lanxess (Lanxess Deutschland GmbH, Germany). Before utilization, the resins were oven-dried overnight at 50 °C. Table 1 lists the characteristics of the chelating resin. Copper sulphate pentahydrate (CuSO_4_·5H_2_O), chromium nitrate nonahydrate (Cr(NO_3_)_3_·9H_2_O), hydrochloric acid (HCl), zinc nitrate hexahydrate (Zn(NO_3_)_2_·6H_2_O), ammonium hydroxide (NH_4_OH), sodium hydroxide (NaOH), calcium sulphate dehydrate (CaSO_4_·2H_2_O), ferric nitrate nonahydrate (Fe(NO_3_)_3_·9 H_2_O), manganese sulphate monohydrate (MnSO_4_·H_2_O), magnesium nitrate hexahydrate (Mg(NO_3_)_2_·6H_2_O), were acquired from Sigma-Aldrich. Aluminum nitrate nonahydrate (Al(NO_3_)_3_·9H_2_O) was obtained from J.T. Baker. The water used throughout the experiments was deionized water (DI). The preparation of metal ion stock solutions involved dissolving measured amounts of the respective metal salts in DI water. Subsequently, appropriate portions of the prepared stock solutions were diluted to prepare the standard working solutions. The pH level of the solutions used were modified by dropwise addition of dilute aqueous NaOH or HCl.

**Table 1:** The characteristics of Lewatit MDS TP 220 chelating resin [18].

Properties	Lewatit MDS TP 220
Appearance	White, opaque
Ionic form	SO_4_^2−^
Resin type	Weakly basic
Structure	Macroporous
Matrix	Polystyrene divinylbenzene
Functional group	Bis-picolylamine
Mean bead size	0.38 ± 0.04 mm
Water retention	50–58
Capacity	36 g/L
pH range	0–14

### 2.2 Characterization

The analysis of the surface morphology of LTP was done via SEM while its elemental composition was determined utilizing energy dispersive spectroscopy (EDS) which was coupled to the SEM (Hitachi SU 3500, Japan). The working parameters included a vacuum pressure of 100 Pa, a voltage of 15 kV, and a varying magnification. The surface functional groups on LTP were analyze based on FTIR using a Frontier spectrometer (PerkinElmer Inc.) equipped with an Attenuated total reflectance (ATR) module. Each recorded spectrum was comprised of four averaged scans captured between 4,000 and 400 cm^−1^ at a resolution of 4 cm^−1^. The zeta potential measurement, including determination of the point of zero charge (pzc) was conducted via an electrokinetic analyzer (SurPASS, Anton Paar GmbH, Austria) utilizing the cylindrical cell technique and 1 mmolL^−1^ potassium chloride (KCl) as electrolyte solution. XPS spectra were measured using PHI Quaterna II scanning XPS microscope (ULVAC-PHI, Physical Electronics, Japan) which is coupled with an Al Kα (1486.6 eV) source. Pristine and Cu(II) loaded LTP resin were used as samples for XPS. The samples were mounted onto a holder using Cu conductive tape (Structure Probe Inc., West Chester, USA) which was sliced into square pieces 1*1 cm (three different spots were analyzed). Auto neutralization by Ar^+^ source was used for all measurements considering insulating nature of the samples. The survey spectra were obtained within the binding energies (BE) range of 0–1100 eV employing an electron pass energy of 224 eV, a speed of 50 ms per step, and a resolution of 0.5 eV per step during seven full sweeps. The high-resolution lines, namely C1s, N1s, O1s, S2p and Cu2p, were recorded in BE ranges (altering with the number of sweeps) of 275.0–295.0 eV (6 sweeps), 388.0–410.0 eV (10 sweeps), 528.0–542.0 (8 sweeps), 165–172 eV (3 sweeps), 927–970 eV (15 sweeps) with pass energy of electrons 20 eV. The quantification of the at-% concentrations of elements was carried out utilizing CasaXPS 2.3.15 (Casa software Ltd., Devon, UK) employing peak areas, as well as the photoionization cross-sections of the elements at the specific photon energy used. The high-resolution spectra of C1s, O1s, N1s, and S2p were deconvoluted using Gaussian/Lorentzian function after subtraction of background by Shirley's method. The Cu2p line was deconvoluted utilizing Gaussian/Lorentzian function after subtraction of U 2 Tougaard background.

### 2.3 Copper adsorption studies

Copper ions adsorption onto LTP was examined in batch mode with varying adsorption conditions including the initial pH, contact time, initial Cu(II) concentration, and temperature. Typically, ∼ 30 mg of LTP was weighed into plastic tubes and 10 mL of prepared Cu(II) solution was added. The contents of the tube were placed on a rotary shaker and agitated for 20 h. Afterwards, the LTP resin was removed from the Cu(II) solution using polypropylene (PP) syringe filter with a porosity of 0.45 μm. Then an inductively coupled plasma mass spectrometer (ICP-MS, Agilent, 7900) was employed to analyze the concentration of Cu(II) in the solutions prior to and after the experiments. Finally, the adsorption efficiency (R, %), and adsorption capacity ($q_{e}$, mg/g) were determined with equations (1), (2)), respectively, provided below [13]:

(1) $$R=\frac{C_{i}-C_{e}}{C_{i}}\times 100\;\%$$

(2) $$q_{e}=\frac{\left(C_{i}-C_{e}\right)V}{m}$$

where m represents the mass of the resin (mg), V is solution volume (mL), $C_{i}$ denotes the initial concentration of metal ion (mg/L) and $C_{e}$ is the equilibrium concentration of metal ion (mg/L).

### 2.4 Copper desorption and reusability studies

The study on Cu(II) desorption from LTP and regeneration of used LTP resins were done subsequent to the completion of the adsorption experiments with 50 mg/L Cu(II) solution. 2 M NH_4_OH was selected as elution solution in this experiment, which is supported by previous research [4,14]. 10 mL of NH_4_OH was pipetted into the tube containing Cu(II)-loaded LTP resins and the contents were agitated for 20 h. Afterwards, the LTP resin was filtered from the elution solution utilizing PP syringe filter with a porosity of 0.45 μm, rinsed with DI water, and subjected to drying for 24-h at 50 °C before proceeding to the subsequent adsorption-desorption cycle. In all. five cycles were carried out and the desorption efficiency (D, %) in each cycle was determined using equation (3) [19]:

(3) $$D\;\left(\%\right)=\frac{C_{d}V_{d}}{C_{i}-C_{e}V}\times 100\;\%$$

where $V_{d}$ is solution volume (mL), and $C_{d}$ represents the Cu(II) concentration (mg/L) in the elution solution.

### 2.5 Selectivity studies

The Cu(II) selectivity of LTP was investigated in the coexistence of other metal ions. Approximately 30 mg of LTP was weighed and placed into plastic tubes and 10 mL of 0.7 mM multi-metal solution were added. The experiment was conducted at pH of 1.5 and 2.5, and the contents of the tube were agitated at room temperature for 20 h with a rotary shaker. Afterwards, the resin was removed from the multi-metal solution and analyzed using ICP. The *q*_*e*_ of the metal ions were calculated using equation (2) given above.

## 3 Results and discussion

### 3.1 LTP characterization

**Fig. 1:**
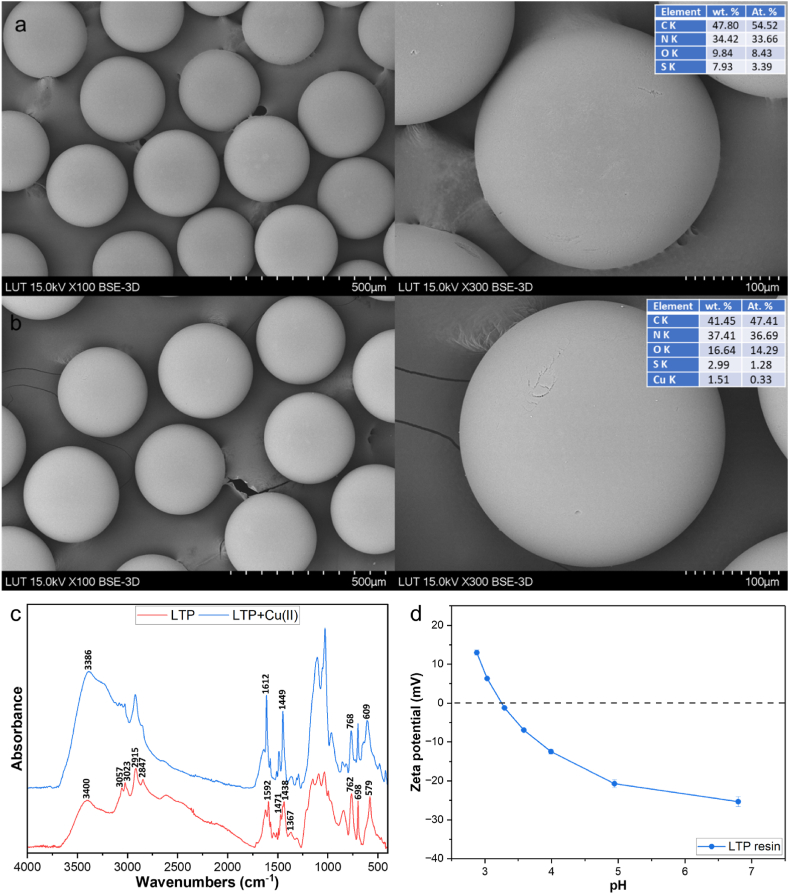
(a) SEM-EDS of LTP pre and (b) post adsorption of Cu(II) (c) FTIR spectra (d) Zeta potential data.

The SEM images and matching EDS data of LTP before and after adsorption of Cu (II) are displayed in Fig. 1a and b. The images revealed the spherical structure and monodispersity of the LTP resin. EDS confirmed the presence of carbon (C, 47.8%), nitrogen (N, 34.4%), and oxygen (O, 9.8%) in the pristine resin, which are coherent with bis-picolylamine functional group (Fig. 1a). The EDS also detected sulphur (S, 7.9%) which confirms the ionic form of the resin. The surface of LTP did not show any significant physical change after adsorption, however, the EDS detected Cu(II) on the resin's surface, along with the previously mentioned elements (Fig. 1b). This confirms successful binding of Cu (II) onto the LTP surface.

Fig. 1c illustrates the FTIR spectra of LTP pre and post Cu(II) adsorption within the wavenumber of 4000-400 cm^−1^. The –OH and N–H stretching vibrations were detected in the 3500−3200 region [20]. The asymmetric stretching of –C–H and –CH_2_ were located at 3057, and at 3023 and 2915 cm^−1^, respectively. The stretch at 2847 cm^−1^ is ascribed to the symmetric stretching vibrations of –CH_2_ [14,21]. The C=N and aromatic skeleton vibrations associated with the pyridine groups were located at 1592 and 1438 cm^−1^, while the amine groups in LTP was corroborated by the bands in the region of 988–1159 cm^−1^ [1,14]. Additionally, the amine groups at 698 cm^−1^, and the bending vibrations of the C–N group and pyridine ring at 579 cm^−1^ and 762 cm^−1^, respectively, were observed [1,[22], [23], [24]]. After Cu(II) adsorption, the intensity of some bands increased while shifts of peaks at 1592, 1438, 762, and 579, to 1612 cm^−1^, 1449, 768 cm^−1^ and 609 cm^−1^, respectively, were detected. This indicates their probable participation in Cu(II) adsorption. More discussion regarding the FTIR spectra of LTP following Cu(II) adsorption is available in section 3.2.5.

The zeta potential of LTP studied across various pH levels are displayed in Fig. 1d. The zeta potential of LTP was observed to decrease gradually with increasing pH ranging from pH 3 to pH 8. The adsorbent's surface charge, which is a result of protonation-deprotonation behavior influences the adsorption processes in terms of efficiency. Simultaneously, the pH of a solution is a key component that influences both the activation of surface charge on the adsorbent and metal speciation. The pzc for LTP was determined to be at pH of 3.25, signifying that the surface charge on LTP becomes negative when the pH is above pzc, encouraging electrostatic interactions [4,14,17]. When pH level is below the pzc, the surface charge on LTP becomes positive, ordinarily resulting in repulsive interactions between Cu(II) and LTP. However, there exist coordinative interactions (chelation) in the case of adsorbents bearing nitrogen, thus countering the repulsive electrostatic force and enabling the capturing of Cu(II) at pH level below pzc [1].

**Fig. 2:**
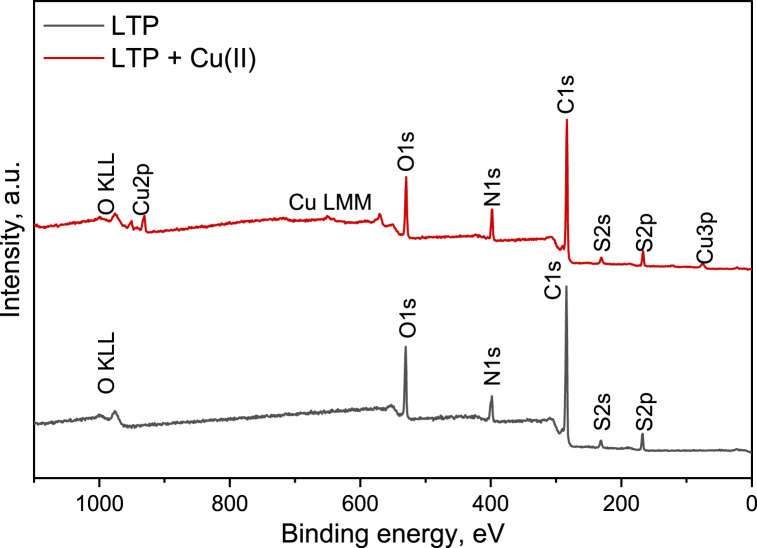
The XPS survey spectra of LTP before and after Cu(II) adsorption.

The survey XPS spectra of pristine and Cu(II) adsorbed samples are displayed in Fig. 2. Five characteristic lines were detected in both samples including S2p (∼166.5 eV), S2s (∼230.3 eV), C1s (284.4 eV), N1s (∼398.6 eV), and O1s (∼528.8 eV). The Cu2p (Cu2p_3/2_–932.0 eV; Cu2p_1/2_–952.0eV) characteristic line in combination with Cu3p (∼75.3 eV) were additionally detected in the XPS spectra of LTP-Cu sample which is evidence of successful adsorption of Cu ions [25]. No peaks related to other transition metals were observed in the XPS spectra of LTP resin after adsorption experiment. The O KLL and Cu LMM Auger lines were detected at ∼975.5 eV and ∼571–719.1 eV [25]. The atomic concentration of elements calculated from survey spectra of the materials are discussed and shown in Fig. S1. The XPS statistical data of LTP, pre and post Cu(II) adsorption are presented in Tables S1 and S2. More information about chemical states of the atoms and bonds was received from analysis of high-resolution spectra of Cu2p, S2p, C1s, N1s, and O1s elements discussed in section 3.2.5.

### 3.2 Adsorption tests

#### 3.2.1 Effect of initial solution pH

**Fig. 3:**
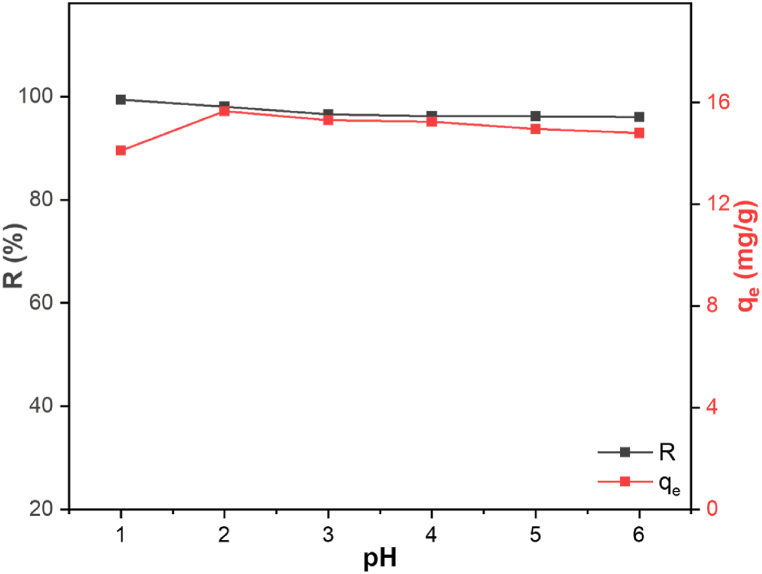
Cu(II) adsorption onto LTP at varying pH levels (condition: 20 h, room temperature, 50 mg/L Cu(II) solution, 3 g/L dosage).

The Cu(II) solution was adjusted from pH 1–6 to investigate its influence on the binding of Cu(II) onto LTP, and the result is presented in Fig. 3. Using 50 mg/L initial concentration, almost 100 % efficiency of Cu(II) uptake was obtained throughout the pH range studied. There was no significant effect of pH on the removal efficiency. The adsorption capacity increased from 14.1 mg/g at pH 1–15.7 mg/g at pH 2, and the capacity remained almost unchanged between pH 2 to pH 6. This corroborates the chelation property of the nitrogen-bearing groups in the LTP resin which reactivity is independent of the pH and surface charge. Similar results were presented by Kołodyńska et al., for Cu(II) adsorption onto M4195 and TP220 [14]. Fig. S2 displays a picture of LTP taken before and after Cu(II) adsorption. The change in color from opaque to blue in the samples post-adsorption indicates the successful binding of Cu(II) onto LTP. Considering that the pH had little or no effect on adsorption capacity, pH 5 was chosen in the ensuing studies with the exception for selectivity experiments, which were carried out at pH 1.5.

#### 3.2.2 Adsorption kinetics

**Fig. 4:**
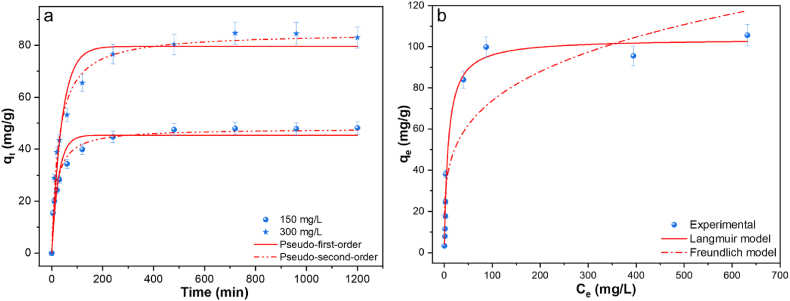
Adsorption of Cu(II) onto LTP at different (a) contact time and (b) initial concentration (condition: 20 h, room temperature, 3 g/L dosage).

The contact time between the Cu(II) solution and LTP was adjusted in the range 0–20 h to study its effect on adsorption. The result shown in Fig. 4a indicates that increasing the contact time increases the amount of Cu(II) adsorbed onto the surface of LTP until equilibrium time. Cu(II) adsorption was fast during the first 5–30 min, primarily driven by the facile interaction between the available binding sites and Cu(II) ions. After 30 min, a slow increase in adsorption rate was observed until equilibrium, due to slower Cu(II) diffusion inside the resin. Adsorption kinetic models were used to further examine the adsorption behavior of Cu(II) on LTP. This was determined by applying the experimental results to the pseudo-first- and pseudo-second-order kinetic models. Equations (4), (5)) given below respectively represent the nonlinear form of both models [26].

(4) $$q_{t}=q_{e}\left(1-\exp\left(-k_{1}t\right)\right)$$

(5) $$q_{t}=\frac{q_{e}^{2}k_{2}t}{{1+q_{e}k}_{2}t}$$

where *q*_*t*_ (mg/g), *k*_*1*_ (min^−1^), *k*_*2*_ (g mg^−1^min^−1^), and *t* (min) denote the adsorption capacity, pseudo-first-order rate constant, pseudo-second-order rate constant, and time, respectively.

The parameters derived from the models and their fitted curves are presented in Table 2 and Fig. 4a, respectively. The pseudo-second-order model which exhibited a correlation coefficient (*R*^*2*^) higher than that of the pseudo-first-order model, appropriately represented the experimental data. This observation signifies that chemical adsorption controls the adsorption process. Similar findings were obtained for Cu(II) adsorption on TP 220 and M 4195 [4,14,17,27].

**Table 2:** The parameters for Cu(II) adsorption onto LTP derived from kinetic models.

Kinetic model	Parameter	300 mg/L	150 mg/L
Pseudo-first-order kinetics	*K*_*1*_ (1/min)	0.026	0.038
	*q*_*e*_ (mg/g)	79.579	45.361
	*R*^*2*^	0.938	0.925
Pseudo-second-order kinetics	*K*_*2*_ (mg/g∙min)	0.0004	0.001
	*q*_*e*_ (mg/g)	84.968	47.959
	*R*^*2*^	0.988	0.980

#### 3.2.3 Adsorption isotherms

LTP adsorption of Cu(II) was studied at concentrations ranging from 0 to 950 mg/L of Cu(II) in solution. The data presented in Fig. 4b indicates that the adsorption capacity increased rapidly with increasing initial Cu(II) concentrations before reaching a plateau at elevated concentrations. This behavior may be due to a greater abundance of binding sites at lower concentrations compared to Cu(II) present in the solution. Higher concentrations, however, resulted in competition for fewer potential binding sites due to the greater Cu(II) in the solution than the number of available sites. This result indicates that LTP can be applied to recover Cu(II) from heavy metal wastewater even in trace concentrations. The experimental results were modeled with the Langmuir and Freundlich isotherm model to estimate the maximum equilibrium uptake and binding affinity. The Langmuir model usually depicts monolayer adsorption onto identical sites on adsorbents’ surface while Freundlich describes mono-and multilayer adsorption on heterogenous sites. The nonlinear form of the Langmuir and Freundlich isotherm models are provided in equations (6), (7)) below [26]:

(6) $$q_{e}=\frac{{q_{m}k_{L}C}_{e}}{1+{k_{L}C}_{e}\;}$$

(7) qe=kFCe1n

where the maximum adsorption capacity of the adsorbent is represented by *q*_*m*_ (mg/g), whereas the Langmuir constant, *k*_*L*_ (L/mg), indicates the adsorption affinity. The adsorbent intensity and capacity are described by the Freundlich constants, *n* and *k*_*F*_ (L/g), respectively.

The parameters obtained from the above models and their fitted curves are given in Table 3 and Fig. 4b, respectively. The Langmuir isotherm model adequately represented the isotherm data, exhibiting a higher *R*^*2*^ than the Freundlich isotherm model. This suggests that the adsorption of Cu(II) onto LTP was by homogenous monolayer attachment as commonly described by the Langmuir model. The maximum Cu(II) adsorption capacity estimated by the Langmuir model was 103.9 mg/g. Table 4 compares the maximum adsorption capacity of LTP to those of alternative bis-picolylamine chelating resins. Additionally, the favorability of the adsorption process was assessed by employing the Langmuir separation factor constant and equilibrium parameter, R_L_ given in equation (8) [5,13].

(8) $$R_{L}=\frac{1}{1+K_{L}\times C_{i}}$$

where an adsorption process can be considered either linear (with R_L_ = 1), irreversible (with R_L_ = 0), favorable (with R_L_ < 1), or unfavorable (with R_L_ > 1). The R_L_ for LTP ranged from 0.006 to 0.363, indicating the favorable nature of Cu(II) adsorption onto LTP.

**Table 3:** The parameters for Cu(II) adsorption onto LTP derived from isotherm models.

Langmuir parameter			Freundlich parameters		
*K*_*L*_ (L/mg)	*q*_*m*_ (mg/g)	*R*^*2*^	*n*	*K*_*F*_ (L/mg)	*R*^*2*^
0.121	103.908	0.978	3.962	23.088	0.820

**Table 4:** A comparison of bis-picolylamine chelating resins for Cu(II) removal.

Resin	pH	*q*_*m*_ (mg/g)	*K*_*L*_ (L/mg)	dosage (g/L)	Time (h)	References
Lewatit monoplus TP220	2	94.20	0.003	10	4	[14]
Dowex M4195	2	56.66	0.003	10	4	[14]
MTS9600	2	43.86	0.063	400	3	[28]
XUS 43578	5	166.30	1.480	–	3	[29]
Dowex M4195	2	101.17	0.414	1	–	[27]
Dowex M4195	1.5	91.51	9.361	0.4	48	[1]
PMMA-PD	1.5	98.18	10.636	0.4	48	[1]
PMMA-PD	1.5	97.03	5.456	0.4	48	[1]
Lewatit MDS TP220	5	103.91	0.121	3	20	This study

#### 3.2.4 Adsorption thermodynamic

The impact of temperature on adsorption was assessed by varying the temperature during experiment between 298 and 328 K. The thermodynamic data, including the changes in entropy (ΔS^0^), enthalpy (ΔH^0^), and Gibbs free energy (ΔG^0^), were obtained using equations (9), (10), (11) below [30]:

(9) $$K_{D}=\frac{q_{e}}{C_{e}}$$

(10) $$\ln\;K_{D}=-\frac{{\Delta H}^{0}}{RT}+\frac{{\Delta S}^{0}}{R}$$

(11) $${\Delta G}^{0}={\Delta H}^{0}-T{\Delta S}^{0}$$

where K_D_ and T symbolize the thermodynamic equilibrium constant (L/g), and temperature (K), while R denote the universal gas constant (8.314 J mol^−1^ K^−1^). The obtained thermodynamic parameters, which describe the kind of the adsorption process, are listed in Table 5 and a plot of *In K*_*D*_ vs I/*T* is displayed in Fig. S3. The positive ΔH^0^ and ΔS^0^ values suggest endothermic adsorption process, and an increased randomness during Cu(II) fixation onto the active sites of LTP at the solid-liquid interface, respectively. All the ΔG^0^ values were negative, which signifies that the adsorption process was spontaneous. Moreover, the decline in ΔG^0^ as temperature rises signifies that higher temperatures favor adsorption [16,31,32]. Nonetheless, the marginal changes observed in ΔG^0^ values as temperature varies suggests that the impact of temperature on the adsorption remains minimal.

**Table 5:** The parameters for adsorption of Cu(II) onto LTP obtained from thermodynamics.

T (K)	ΔS^0^ (J mol^−1^K^−1^)	ΔH^0^ (kJ mol^−1^)	ΔG^0^ (kJ mol^−1^)
298	27.97	2.76	−5.57
318			−6.13
328			−6.41

#### 3.2.5 Analysis of adsorption mechanism

The characterization results from FTIR revealed significant shifts towards higher wavenumbers and increased intensity in some bands after Cu(II) adsorption. The shifts of bands at 1591, 1438, 761, and 579, to 1612 cm^−1^, 1449, 768 cm^−1^ and 609 cm^−1^, respectively. The observed shifts subsequent to the binding of Cu(II) onto LTP are similarly associated to the vibrations originating from the pyridine and aliphatic amine functional groups, suggesting their involvement in the binding of Cu(II). These changes in vibrational peaks can be attributed to changes in the coordination environment coordination environment around specific functional groups on the resin's surface. For instance, the formation of strong Cu-bispicolylamine complexes often induces changes in the vibrational modes of the chelating groups to higher wavenumbers. This phenomenon is consistent with findings in the literature [1,4,29,33,34]. On the other hand, increased intensity in certain bands can be ascribed to the strengthening of chemical bonds upon Cu(II) adsorption [35]. This discernible increase in intensity strongly implies a more substantial interaction, leading to leading to the formation of exceptionally strong complexes between the chelating resin and Cu(II). The asymmetric nature of bonds within bis-picolylamine groups makes them especially responsive to coordination with Cu(II) ions. Changes in bond strengths and local environments around the asymmetric bonds, particularly those involving nitrogen atoms with asymmetrically distributed lone pairs, play a crucial role in altering vibrational modes and significantly contribute to shifts in band positions.

**Fig. 5:**
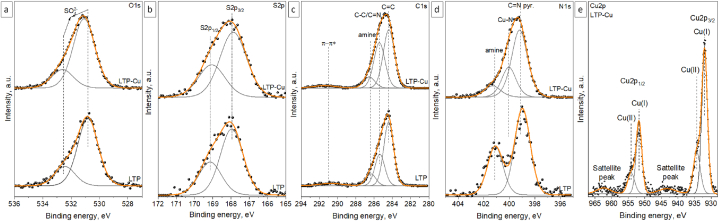
High-resolution spectra of (a) O1s, (b) S2p, (c) C1s, (d) N1s, (e) Cu2p lines measured for resin samples before and after adsorption.

Furthermore, the high-resolution spectra of C1s, N1s, S2p O1s, Cu2p lines for samples before and after adsorption are presented in Fig. 5a–e. There are no significant changes in O1s and S2p spectra of LTP before and after adsorption (Fig. 5a and b). The position of peaks in O1s spectra are typical for oxygen atoms in SO4^2−^ anion as well as position of doublet of peaks in S2p spectra. The small shift of the oxygen peak after adsorption experiment could be the result of either change in oxygen atoms environment (e.g., effect of Cu(II) adsorption) or simply insulating nature of the sample. The C1s spectra of the samples (Fig. 5c) were deconvoluted using four peaks. The peak at BE ∼284.4 eV is related to sp^2^-hybrid carbon bonds including C=C bonds from pyridine groups in bis-picolylamine and polystyrene divinylbenzene backbone [36]. The peak at BE ∼285.4 eV is assigned to sp^3^-hybrid carbon atoms in C–C bonds from polystyrene divinylbenzene backbone [36] and C–N bonds in amine from bis-picolylamine, as well as sp^2^-hybrid carbon atoms in C=N bonds from pyridine groups [37]. The peak at BE ∼286.5 eV is attributed to the bonds of C–N in the amine group. The peak at BE ∼291 eV is the result of secondary process and is assigned to π→π* shake-up satellite [38]. The only difference in C1s spectra of the samples is area of the peak related to C=N and C–C bonds. This is in line with the analysis of survey XPS spectra and FTIR spectra which showed higher concentrations of nitrogen in sample and higher intensity of C=N bond (1610 cm^−1^), respectively. The N1s spectra of the pristine resin ((Fig. 5d) is described by two peaks. The first peak is situated at BE ∼399.2 eV and is allocated to the bonds of C=N in the pyridine ring, while the second peak at BE ∼401.2 eV is characteristic for substituted amines [39,40]. The new peak at BE ∼400.1 eV appears in N1s spectra after Cu adsorption. In Bulushev et al. peak related to formation of Cu–N bond between pyridinic nitrogen embedded in structure of carbon materials and Cu is located at 399 eV which overlaps with peak observed in the present work [41]. Considering insulating nature of resin investigated in this work, the shift of this peak in 1 eV is reasonable. Moreover, in Clarke et al., 2005, it was shown that a shift of the N1s line by 0.8–1.2 eV happen after coordination of Cu ions by 1,10-phenantroline which can be used as reference system to some extent [42]. Despite that, significant change of C=N pyridine/amine bonds ratio happened after adsorption. Decreasing of the amine peak area can happen due to amine group dehydrogenation with formation of additional C=N groups. Thus, in Sarmiento-Pavía et al., it has been shown that such amine ligands as 2,10-bis-(2′-pyridyl)-3,6,9-triazundecane can be dehydrogenated by action of Cu(II) salts under the exclusion of O_2_ with formation of additional C=N group and possible reduction of Cu(II) to Cu(I) [43]. Notwithstanding that bis-picolylamine contains only three nitrogen centers (two from pyridine rings and one substituted amine group), it is still possible that similar reaction happened. Moreover, the ordinary position of C=N group is 399 eV [44], but there is no information about effect of Cu coordination on BE shift. Thus, peak can be shifted for C=N group formed during Cu(II) coordination and subsequent dehydrogenation reaction toward higher BE by 0.8–1.2 eV. The deconvolution of Cu2p spectra (Fig. 5e) confirmed reduction of Cu(II) to Cu(I). Thus, first doublet of peaks located at BE ∼932 eV (Cu2p_3/2_) and BE∼ 951.8 eV (Cu2p_1/2_) is associated to the diamagnetic Cu(I), which is additionally confirmed by absence of intense satellite peak [38,41]. But the presence of satellite lines with low intensity is an evidence of presence of small amount of paramagnetic Cu(II). Thus, the second doublet of peaks located at BE ∼934.4 eV and ∼935.1 eV relates to Cu(II) [38,41].

#### 3.2.6 Cu(II) desorption and reusability of LTP

**Fig. 6:**
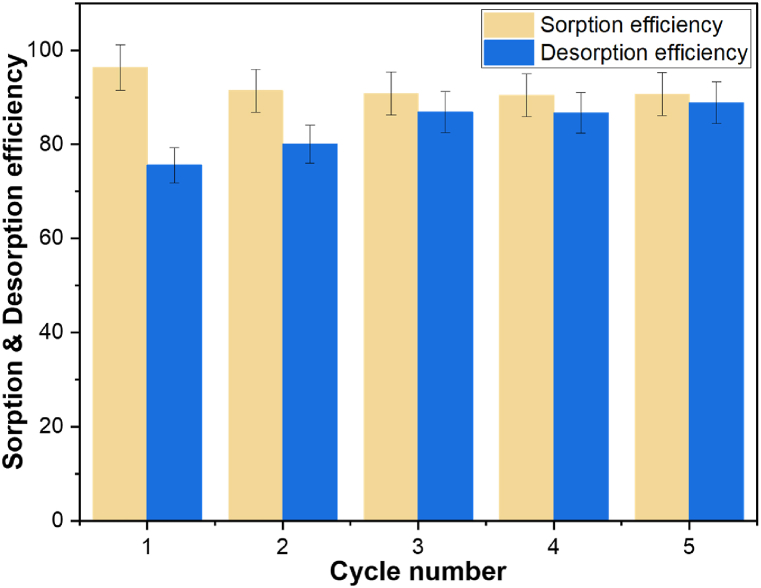
LTP desorption and reusability across five cycles (conditions: room temperature, 20 h, 50 mg/L Cu(II) solution, 3 g/L dosage).

In adsorption processes, the possibility of recovering adsorbed metals and the potential for adsorbent recycling holds significant importance [31]. The recovery of Cu(II) and the reusability of LTP was evaluated through five successive adsorption-desorption cycles. The outcomes, as depicted in Fig. 6 illustrates a slight decrease in the adsorption efficiency of LTP from 96 to 91% after the first cycle, then the efficiency stayed at *∼*91% in the remaining cycles. Conversely, the desorption efficiency increased from of 76–89% across these cycles, signifying successful recovery of substantial amount of adsorbed Cu(II) from the adsorbent. This result shows the potential of the adsorbent for recurring reuse for the recovery of Cu(II).

#### 3.2.7 Selectivity studies

**Fig. 7:**
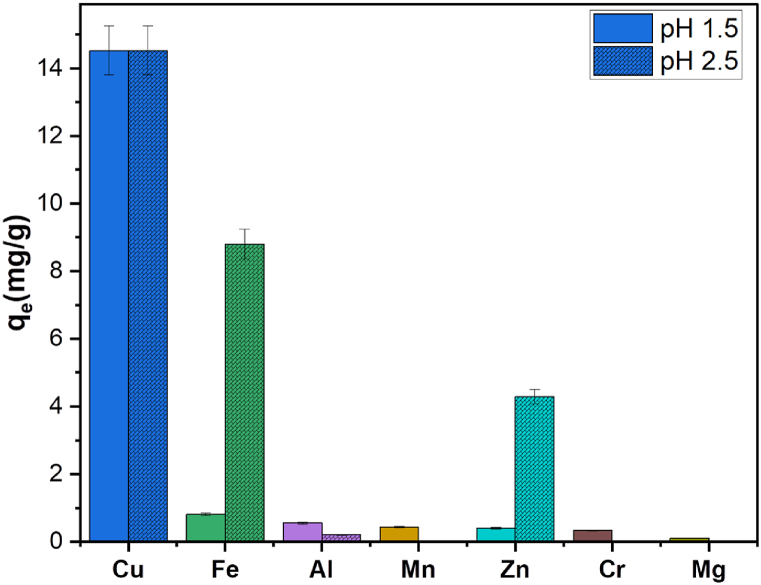
Selectivity of LTP towards Cu(II) in a multi-metal solution (condition: 20 h, room temperature, 3 g/L dosage, 0.7 mM).

The selective adsorption of Cu(II) from a multi-metal solution onto LTP was studied. The multi-metal solution comprised Al(III), Zn(II), Mn(II), Cr(III), Fe(III), Mg(II), and Ca(II) representing metals ions commonly coexisting with Cu(II) in wastewater and CTs [2,45]. The data presented in Fig. 7 reveals the high selectivity of LTP towards Cu(II) in relation to the other metals at pH 1.5. While the Cu(II) uptake was 14.9 mg/g at the pH, the uptake for the remaining metal ions was nearly insignificant, falling lower than 0.85 mg/g. However the selectivity for Cu(II) slightly decline at pH 2.5 and more Fe(III) and Zn(II) were adsorbed. The indicates that more acidic pH favors LTP selectivity for Cu(II). The high selectivity of LTP towards Cu(II) can be attributed to the strong affinity of chelating polymers bearing solely nitrogen donor atoms towards Cu(II), especially in strongly acidic conditions [14]. Additionally, copper displays higher absolute electronegativity and greater distribution coefficient, which results in formation of more stable complexes compared to the coexisting metal ions [26,46,47].

## 4 Conclusion

The binding capacity, kinetics, thermodynamics and selectivity of Lewatit MDS TP 220 chelating resin for Cu(II) has been studied in this work. SEM analysis revealed that the resin possessed a spherical and monodisperse morphology, with EDS confirming the presence of Cu(II) on LTP's surface post-adsorption. The FTIR spectra indicated the presence of aliphatic amine and pyridine group, which corresponds to the characteristics of bis-picolylamine groups. The adsorption isotherm and kinetics could be described by Langmuir isotherm and pseudo-second-order kinetic models, respectively. This suggests that the binding of Cu(II) onto LTP occurred through a monolayer process involving chemical interactions. The resin showed fast adsorption kinetics within the initial 5−30 min and *q*_*m*_ of 103.9 mg/g. The adsorption thermodynamics revealed that it was a spontaneous and endothermic process. FTIR and XPS studies suggested coordination or chelation as the possible adsorption mechanism. Lewatit MDS TP 220 demonstrated excellent Cu(II) adsorption, desorption with 2 M NH_4_OH, and reusability after 5 regeneration steps. Furthermore, it exhibited high selectivity in multi-metal solution with a pH of 1.5, owing to its strong affinity for Cu(II). This study shows the potential of the LTP resin for effective and recurring Cu(II) recovery from Cu tailings and wastewater, thus contributing to decontamination of the environment.

## Funding

The funding for this research was provided by 10.13039/501100014438Business Finland under the project 5R REFINERY (grant number 43303/31/2020).

## Data availability statement

Data will be made available on request.

## CRediT authorship contribution statement

**Kosisochi Ibebunjo:** Writing – original draft, Visualization, Methodology, Investigation, Conceptualization. **Youssef El Ouardi:** Writing – review & editing, Supervision, Methodology. **John Kwame Bediako:** Writing – review & editing, Supervision. **Anna Iurchenkova:** Writing – original draft, Investigation. **Eveliina Repo:** Writing – review & editing, Visualization, Supervision, Project administration, Funding acquisition.

## Declaration of competing interest

The authors declare that they have no known competing financial interests or personal relationships that could have appeared to influence the work reported in this paper.

## References

[1] Liu Z., Wang L., Lv Y., Xu X., Zhu C., Liu F., Li A. (2021). Impactful modulation of micro-structures of acid-resistant picolylamine-based chelate resins for efficient separation of heavy metal cations from strongly acidic media. Chem. Eng. J..

[2] Ryu S., Naidu G., Moon H., Vigneswaran S. (2019). Selective copper extraction by multi-modified mesoporous silica material, SBA-15. Sci. Total Environ..

[3] Yan H., Dai J., Yang Z., Yang H., Cheng R. (2011). Enhanced and selective adsorption of copper(II) ions on surface carboxymethylated chitosan hydrogel beads. Chem. Eng. J..

[4] Wołowicz A., Hubicki Z. (2020). Enhanced removal of copper(II) from acidic streams using functional resins: batch and column studies. J. Mater. Sci..

[5] Gao J., Zhang L., Liu S., Liu X. (2022). Enhanced adsorption of copper ions from aqueous solution by two-step DTPA-modified magnetic cellulose hydrogel beads. Int. J. Biol. Macromol..

[6] Suwannahong K., Sripirom J., Sirilamduan C., Thathong V., Kreetachart T., Panmuang P., Deepatana A., Punbut S., Wongcharee S. (2022). Selective chelating resin for copper removal and recovery in aqueous acidic solution generated from synthetic copper-citrate complexes from bioleaching of E-waste. Adsorpt. Sci. Technol..

[7] Tapaswi P.K., Moorthy M.S., Park S.S., Ha C.S. (2014). Fast, selective adsorption of Cu2+ from aqueous mixed metal ions solution using 1,4,7-triazacyclononane modified SBA-15 silica adsorbent (SBA-TACN). J. Solid State Chem..

[8] Lü C., Wang Y., Qian P., Liu Y., Fu G., Ding J., Ye S., Chen Y. (2018). Separation of chalcopyrite and pyrite from a copper tailing by ammonium humate. Chinese J. Chem. Eng..

[9] Wang J., Zhang M., Zhou R., Li J., Zhao W., Chen W., Zeng J. (2020). Application of copper tailings combined with persulfate for better removing methyl orange from wastewater. Water Sci. Technol..

[10] Zhou R., Zhang M. (2022). Novel scheme for synergistic purification of copper mine tailings and orthophosphate. Water Sci. Technol..

[11] Statista Research Department (2023). https://www.statista.com/statistics/1353344/global-annual-tailings-production-from-copper-mining/.

[12] ICMM (2022). https://www.icmm.com/en-gb/guidance/innovation/2022/tailings-reduction-roadmap.

[13] Liu J., Zhou R., Yu J., Guo L., Li X., Xiao C., Hou H., Chi R., Feng G. (2022). Simultaneous removal of lead, manganese, and copper released from the copper tailings by a novel magnetic modified biosorbent. J. Environ. Manage..

[14] Kołodyńska D., Sofińska-Chmiel W., Mendyk E., Hubicki Z. (2014). DOWEX M 4195 and LEWATIT®MonoPlus TP 220 in heavy metal ions removal from acidic streams. Sep. Sci. Technol..

[15] Wołowicz A., Hubicki Z. (2012). The use of the chelating resin of a new generation Lewatit MonoPlus TP-220 with the bis-picolylamine functional groups in the removal of selected metal ions from acidic solutions. Chem. Eng. J..

[16] Shen C., Chang Y., Fang L., Min M., Xiong C.H. (2016). Selective removal of copper with polystyrene-1,3-diaminourea chelating resin: synthesis and adsorption studies. New J. Chem..

[17] Neto I.F.F., Sousa C.A., Brito M.S.C.A., Futuro A.M., Soares H.M.V.M. (2016). A simple and nearly-closed cycle process for recycling copper with high purity from end life printed circuit boards. Sep. Purif. Technol..

[18] (2017). Lanxess Product Data Sheet.

[19] Zhang D., Xiao J., Guo Q., Yang J. (2019). 3D-printed highly porous and reusable chitosan monoliths for Cu(II) removal. J. Mater. Sci..

[20] Sofińska-Chmiel W., Kołodyńska D. (2018). Application of ion exchangers for the purification of galvanic wastewater from heavy metals. Sep. Sci. Technol..

[21] Kołodyńska D., Hałas P., Michalski R. (2019). Development of ion exchangers for the removal of health-hazardous perchlorate ions from aqueous systems. Appl. Geochemistry..

[22] Wołowicz A., Hubicki Z. (2018). Comparison of ion-exchange resins for efficient cobalt(II) removal from acidic streams. Chem. Eng. Commun..

[23] Kanagathara N., Marchewka M.K., Drozd M., Renganathan N.G., Gunasekaran S., Anbalagan G. (2013). FT-IR, FT-Raman spectra and DFT calculations of melaminium perchlorate monohydrate. Spectrochim. Acta Part A Mol. Biomol. Spectrosc..

[24] Xu G., Xie Y., Cao J., Tao M., Zhang W.Q. (2016). Highly selective and efficient chelating fiber functionalized by bis(2-pyridylmethyl)amino group for heavy metal ions. Polym. Chem..

[25] Moulder J.F., Stickle W.F., Sobol P.E., Bomben K.D. (1992). Handbook of X-ray photoelectron spectroscopy: a reference book of standard spectra for identification and interpretation of Xps data.

[26] Bediako J.K., Kang J.H., Yun Y.S., Choi S.H. (2022). Facile processing of polyelectrolyte complexes for immobilization of heavy metal ions in wastewater. ACS Appl. Polym. Mater..

[27] Gao J., Liu F., Ling P., Lei J., Li L., Li C., Li A. (2013). High efficient removal of Cu(II) by a chelating resin from strong acidic solutions: complex formation and DFT certification. Chem. Eng. J..

[28] Ulloa L., Bringas E., San-Román M.F. (2020). Simultaneous separation of nickel and copper from sulfuric acid using chelating weak base resins. J. Chem. Technol. Biotechnol..

[29] Edebali S., Pehlivan E. (2016). Evaluation of chelate and cation exchange resins to remove copper ions. Powder Technol..

[30] El Ouardi Y., Lamsayah M., Butylina S., Geng S., Esmaeili M., Giove A., Massima Mouele E.S., Virolainen S., El Barkany S., Ouammou A., Repo E., Laatikainen K. (2022). Sustainable composite material based on glutenin biopolymeric-clay for efficient separation of rare earth elements. Chem. Eng. J..

[31] Xiong C., Chen X., Liu X. (2012). Synthesis, characterization and application of ethylenediamine functionalized chelating resin for copper preconcentration in tea samples. Chem. Eng. J..

[32] Elfeghe S., Anwar S., James L., Zhang Y. (2022). Adsorption of Cu(II) ions from aqueous solutions using ion exchange resins with different functional groups. Can. J. Chem. Eng..

[33] Suwannahong K., Sirilamduan C., Deepatana A., Kreetachat T., Wongcharee S. (2022). Characterization and optimization of polymeric bispicolamine chelating resin: performance evaluation via RSM using copper in acid liquors as a model substrate through ion exchange method. Molecules.

[34] Hsu J.C., Huang H.C., Liu C.Y. (1993). A new selective and high affinity polymeric complexing agent for transition metal ions. Transit. Met. Chem..

[35] Malik H., Qureshi U.A., Muqeet M., Mahar R.B., Ahmed F., Khatri Z. (2018). Removal of lead from aqueous solution using polyacrylonitrile/magnetite nanofibers. Environ. Sci. Pollut. Res..

[36] Merche D., Hubert J., Poleunis C., Yunus S., Bertrand P., De Keyzer P., Reniers F. (2010). One step polymerization of sulfonated polystyrene films in a dielectric barrier discharge. Plasma Process. Polym..

[37] Gabka G., Bujak P., Giedyk K., Kotwica K., Ostrowski A., Malinowska K., Lisowski W., Sobczak J.W., Pron A. (2014). Ligand exchange in quaternary alloyed nanocrystals - a spectroscopic study. Phys. Chem. Chem. Phys..

[38] Briggs D. (2005).

[39] Ravi S., Zhang S., Lee Y.R., Kang K.K., Kim J.M., Ahn J.W., Ahn W.S. (2018). EDTA-functionalized KCC-1 and KIT-6 mesoporous silicas for Nd3+ ion recovery from aqueous solutions. J. Ind. Eng. Chem..

[40] Large A.I., Wahl S., Abate S., da Silva I., Jaen J.J.D., Pinna N., Held G., Arrigo R. (2020). Investigations of carbon nitride-supported mn3 o4 oxide nanoparticles for orr. Catalysts.

[41] Bulushev D.A., Chuvilin A.L., Sobolev V.I., Stolyarova S.G., Shubin Y.V., Asanov I.P., Ishchenko A.V., Magnani G., Riccò M., Okotrub A.V., Bulusheva L.G. (2017). Copper on carbon materials: stabilization by nitrogen doping. J. Mater. Chem. A..

[42] Clarke R., Latham K., Rix C., Hobday M., White J. (2005). Novel copper materials based on the self-assembly of organophosphonic acids and bidentate amines. CrystEngComm.

[43] Sarmiento-Pavía P.D., Flores-Álamo M., Solano-Peralta A., Kroneck P.M.H., Sosa-Torres M.E. (2018). Copper (II)-mediated oxidative dehydrogenation of amine ligands. Inorg. Chim. Acta..

[44] Kehrer M., Duchoslav J., Hinterreiter A., Cobet M., Mehic A., Stehrer T., Stifter D. (2019). XPS investigation on the reactivity of surface imine groups with TFAA. Plasma Process. Polym..

[45] Urrutia C., Yañez-Mansilla E., Jeison D. (2019). Bioremoval of heavy metals from metal mine tailings water using microalgae biomass. Algal Res..

[46] Bediako J.K., Wei W., Kim S., Yun Y.S. (2015). Removal of heavy metals from aqueous phases using chemically modified waste Lyocell fiber. J. Hazard Mater..

[47] Li L., Liu F., Jing X., Ling P., Li A. (2011). Displacement mechanism of binary competitive adsorption for aqueous divalent metal ions onto a novel IDA-chelating resin: isotherm and kinetic modeling. Water Res..

