# Supplementary material for: Selective recovery of copper from copper tailings and wastewater using chelating resins with bis-picolylamine functional groups
Source: Heliyon. 2024 Mar 12;10(6):e27766. doi: 10.1016/j.heliyon.2024.e27766 (PMC10955294; doi:10.1016/j.heliyon.2024.e27766)
Supplement: Multimedia component 1 [file mmc1.docx]

**Selective recovery of copper from copper tailings and wastewater using chelating resins with bis-picolylamine functional groups**

Kosisochi Ibebunjo^a^**^*^**, Youssef El Ouardi^a^, John Kwame Bediako^a^, Anna Iurchenkova^b^, and Eveliina Repo^a^

^a^School of Engineering Science, Department of Separation Science, LUT University, FI-53850 Lappeenranta, Finland.

^b^Uppsala University, Disciplinary Domain of Science and Technology, Technology, Department of Materials Science and Engineering, Nanotechnology and Functional Materials.

**Supplementary data**

The atomic concentration of elements calculated from survey spectra of the materials are shown in Fig. S1 (1,2). The LTP resin mainly consist of carbon, oxygen, and nitrogen atoms. The surface concentration of carbon and oxygen remains almost the same before (~ 77.2 at. % of C and ~ 11.2 at. % of O) and after Cu(II) adsorption (~ 75.9 at. % of C and ~ 10.3 at. % of O), while concentration of nitrogen increases from 6.8 at. % to 9.0 at. % after adsorption experiment. The same phenomena were observed by FTIR which showed increasing of C=N characteristic bond after the adsorption experiment. It could be either related to the complexation of Cu(II) by the pyridinic group which results in increasing of N content in area of analysis. The concentrations of oxygen and sulphur remain the same before (~ 11.2 at. % at. % of O and ~ 4.8 at. % of S) and after adsorption (~ 10.5 at. % at. % of O and ~ 4.6 at. % of S). The deviation of O/S ratio from stoichiometry of SO_4_^2-^ can happen due to the different stability of bonds under electron beam and Ar^+^ neutralization gun. From the other point of view, such an insulating polymer as bis-picolylamine has low stability under electron beam and Ar^+^ neutralization which can cause radiational damage and/or sputtering of surface that significantly affect ratio of elements because of their different sputtering rate (the lighter elements, the faster sputtering) and/or surrounding (different chemical bonds have different stability). Thus, lower concentration of nitrogen before adsorption can be either result of sputtering of the nitrogen from surface during analysis, while complexation of Cu stabilizes nitrogen atoms because of chemical bonding (high affinity of Cu to N) and prevent sputtering of nitrogen. This likely led to the higher content of detected nitrogen after Cu(II) adsorption. The surface concentration of Cu after adsorption reaches 0.34 at. %. The statistical data measured in five different points of the materials are shown in Tables S1 and S2, providing information about homogeneity of the samples. According to the data, the difference in concentration of elements in different points is ~ 1%.

Fig. S1. Atomic concentration of elements calculated from survey spectra of LTP before and after Cu(II) adsorption.

Table S1. XPS statistical data of LTP (before Cu(II) adsorption).

| LTP | 1st | 2nd | 3rd | 4th | 5th | Sum |  |
| --- | --- | --- | --- | --- | --- | --- | --- |
| C | 77.72 | 77.84 | 76.50 | 77.04 | 76.63 | 77.15 | 0.55 |
| O | 10.88 | 11.15 | 11.58 | 11.17 | 11.42 | 11.24 | 0.24 |
| N | 6.82 | 6.40 | 7.03 | 6.95 | 6.98 | 6.84 | 0.23 |
| S | 4.58 | 4.60 | 4.89 | 4.83 | 4.97 | 4.77 | 0.16 |
| C/N | 11,40 | 12,16 | 10,88 | 11,08 | 10,98 | 11,30 | 0,46 |
| C/O | 7,14 | 6,98 | 6,61 | 6,90 | 6,71 | 6,87 | 0,19 |
| C/S | 16,97 | 16,92 | 15,64 | 15,95 | 15,42 | 16,18 | 0,65 |
| N/S | 1,49 | 1,39 | 1,44 | 1,44 | 1,40 | 1,43 | 0,03 |
| O/S | 2,38 | 2,42 | 2,37 | 2,31 | 2,30 | 2,35 | 0,05 |

Table S2. XPS statistical data of LTP-Cu (after copper adsorption).

| LTP-Cu | 1st | 2nd | 3rd | 4th | 5th | Sum |  |
| --- | --- | --- | --- | --- | --- | --- | --- |
| C | 75,78 | 75,91 | 75,16 | 77,40 | 75,23 | 75,90 | 0,81 |
| O | 10,55 | 9,95 | 10,57 | 10,08 | 10,50 | 10,33 | 0,26 |
| N | 8,81 | 9,28 | 9,09 | 8,30 | 9,22 | 8,94 | 0,36 |
| Cu | 0,31 | 0,34 | 0,35 | 0,26 | 0,41 | 0,33 | 0,05 |
| S | 4,55 | 4,52 | 4,83 | 3,96 | 4,63 | 4,50 | 0,29 |
| C/N | 8,60 | 8,18 | 8,27 | 9,33 | 8,16 | 8,49 | 0,44 |
| C/O | 7,18 | 7,63 | 7,11 | 7,68 | 7,16 | 7,35 | 0,25 |
| C/S | 16,65 | 16,79 | 15,56 | 19,55 | 16,25 | 16,87 | 1,36 |
| N/S | 1,94 | 2,05 | 1,88 | 2,10 | 1,99 | 1,99 | 0,08 |
| O/S | 2,32 | 2,20 | 2,19 | 2,55 | 2,27 | 2,30 | 0,13 |


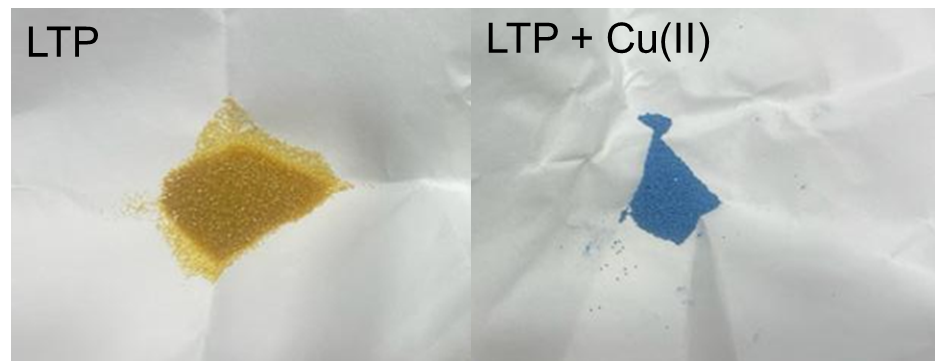


Figure S2. LTP before and after Cu(II) adsorption.

Figure S3. Adsorption of Cu(II) onto LTP at different temperatures.

**References**

1. Yeh JJ, Lindau I. Atomic subshell photoionization cross sections and asymmetry parameters: 1 ⩽ Z ⩽ 103. Acad Press Inc. 1985;32:1–155.

2. Moulder JF, Stickle WF, Sobol PE, Bomben KD. Handbook of X-ray Photoelectron Spectroscopy: A Reference Book of Standard Spectra for Identification and Interpretation of Xps Data. Physical Electronics; 1992. 1–261 p.
